# The genetics of human hematopoiesis and its disruption in disease

**DOI:** 10.15252/emmm.201910316

**Published:** 2019-07-17

**Authors:** Erik L Bao, Aaron N Cheng, Vijay G Sankaran

**Affiliations:** ^1^ Division of Hematology/Oncology Boston Children's Hospital Harvard Medical School Boston MA USA; ^2^ Department of Pediatric Oncology Dana‐Farber Cancer Institute Harvard Medical School Boston MA USA; ^3^ Broad Institute of MIT and Harvard Cambridge MA USA; ^4^ Harvard‐MIT Health Sciences and Technology Harvard Medical School Boston MA USA; ^5^ Harvard Stem Cell Institute Cambridge MA USA

**Keywords:** blood disorders, genetics, genome‐wide association studies, hematopoiesis, Genetics, Gene Therapy & Genetic Disease, Haematology

## Abstract

Hematopoiesis, or the process of blood cell production, is a paradigm of multi‐lineage cellular differentiation that has been extensively studied, yet in many aspects remains incompletely understood. Nearly all clinically measured hematopoietic traits exhibit extensive variation and are highly heritable, underscoring the importance of genetic variation in these processes. This review explores how human genetics have illuminated our understanding of hematopoiesis in health and disease. The study of rare mutations in blood and immune disorders has elucidated novel roles for regulators of hematopoiesis and uncovered numerous important molecular pathways, as seen through examples such as Diamond‐Blackfan anemia and the *GATA2* deficiency syndromes. Additionally, population studies of common genetic variation have revealed mechanisms by which human hematopoiesis can be modulated. We discuss advances in functionally characterizing common variants associated with blood cell traits and discuss therapeutic insights, such as the discovery of BCL11A as a modulator of fetal hemoglobin expression. Finally, as genetic techniques continue to evolve, we discuss the prospects, challenges, and unanswered questions that lie ahead in this burgeoning field.

**Glossary**
Cis‐regulatory element: Genomic regions of transcription factor binding sites and other non‐coding DNA that can influence transcription of a nearby gene. Examples include promoters, enhancers, and silencers.
Common variant association studies (CVAS): Genetic studies which aim to identify common variants (usually defined as minor allele frequency > 1%) associated with a phenotype of interest.
Congenital dyserythropoietic anemia type II (CDA II): The most common subtype of a group of rare hereditary disorders characterized by congenital anemia, ineffective erythropoiesis, the development of secondary hemochromatosis, and uniquely among CDA II, an abnormal glycosylation of erythrocyte membrane proteins.
Diamond‐Blackfan anemia (DBA): A rare inherited bone marrow failure syndrome characterized by normochromic macrocytic anemia, limited cytopenias of other lineages, low reticulocytes, and decreased erythroid precursor cells in the bone marrow.
Eosinophil: A type of white blood cell that plays important roles in fighting certain parasitic infections, and is also implicated in conditions such as allergies and asthma.
Epistasis: Interactions between genetic loci in their effect on a trait, such that the impact of a particular genotype depends on the genotype at other loci in a non‐independent manner.
Expression‐quantitative trait locus (eQTL): Associations of DNA sequence variation with changes in gene expression.
Familial platelet disorder with predisposition to myeloid leukemia (FPDMM): A rare inherited blood disorder caused by mutations of the *RUNX1* gene, clinically characterized by low platelet count, abnormal platelet function, and an increased risk of developing other blood disorders or cancers such as myelodysplastic syndrome (MDS) and acute myeloid leukemia (AML).
Fanconi anemia (FA): A heterogeneous genetic syndrome associated with risk of congenital malformations, bone marrow failure, and cancer.
Genome‐wide association study (GWAS): A genetic analysis that tests for genome‐wide associations between genetic variants and a phenotype of interest.
Haploinsufficiency: The phenomenon in which a single functional copy of a gene is insufficient to maintain normal function.
Heritability: The proportion of variation in a particular trait that is attributable to genetic factors.
Imputation: The use of linkage patterns in a more densely sequenced reference panel to predict unobserved genotypes in a study dataset.
Linkage: The nonrandom association of alleles at different loci.
Myelodysplastic syndrome (MDS): A heterogeneous group of malignant hematopoietic stem cell disorders characterized by dysplastic and ineffective blood cell production and a risk of transformation to acute leukemia.
Neutropenia: A decrease in circulating neutrophils.
Pleiotropy: A phenomenon in genetics whereby a DNA mutation or variant has an effect on multiple traits.
Polycythemia: An increased hemoglobin concentration and/or hematocrit in peripheral blood.
Polygenic risk score: A weighted sum of the number of risk alleles for a phenotype carried by an individual, where the risk alleles and their weights are usually defined by association loci and their effect sizes detected from genome‐wide association studies.
Population stratification: Sample structure due to differences in genetic ancestry among samples.
Rare variant association studies (RVAS): Genetic studies which aim to identify rare variants (usually defined as minor allele frequency < 1%) and their effects on a phenotype of interest.
Sickle cell disease (SCD): A monogenic blood disease caused by a glutamic acid to valine substitution in the β‐globin chain of normal adult hemoglobin, which causes polymerization of mutated sickle hemoglobin and deformation of red blood cells under conditions of deoxygenation.
Thrombocytopenia‐absent radius (TAR) syndrome: A rare congenital syndrome primarily characterized by limb anomalies and low platelet counts.
Thrombocytopenia: A low number of platelets in the blood.
β‐Thalassemia: A group of autosomal recessive hereditary anemias characterized by reduced or absent beta‐globin chain synthesis, leading to alpha‐ and beta‐chain imbalances that cause clinical manifestations of hemolytic anemia and impaired iron handling.

## Introduction

**Figure 1:**
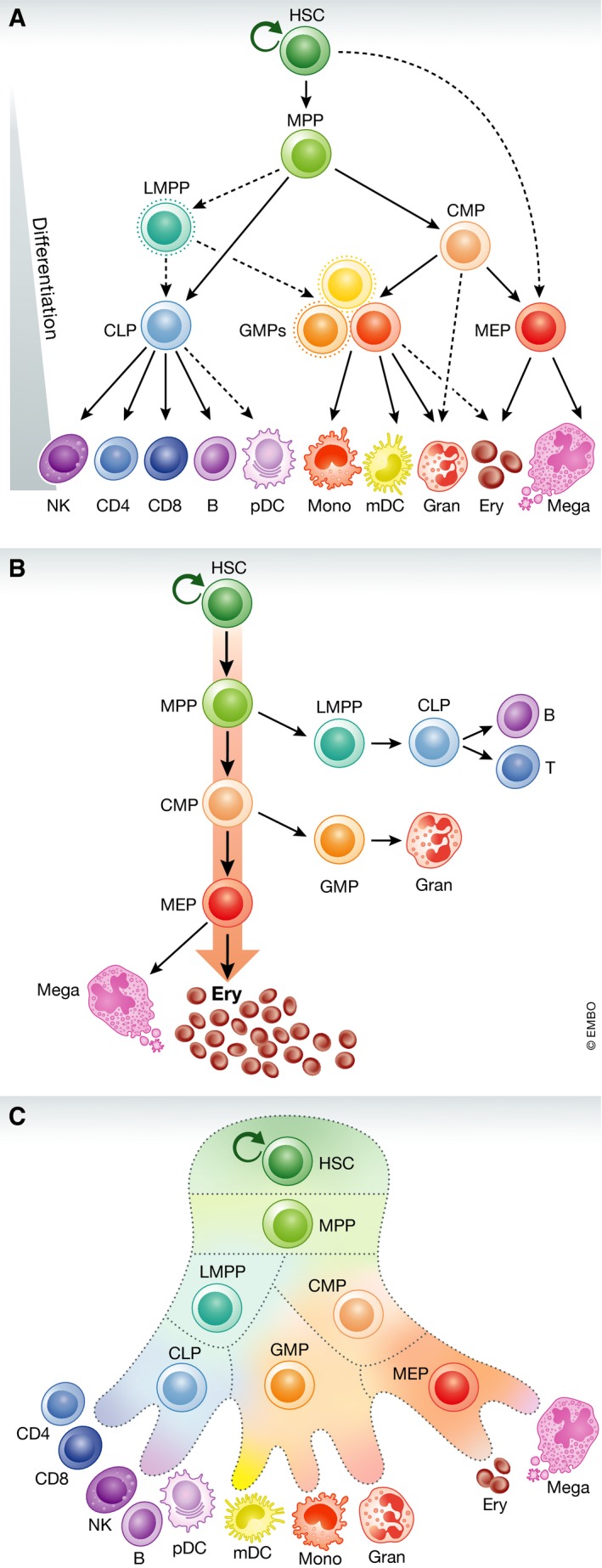
Overview of hematopoiesis (A) Schematic of the human hematopoietic hierarchy. Dashed lines indicate recently discovered differentiation paths. mono, monocyte; gran, granulocyte; ery, erythroid; mega, megakaryocyte; CD4, CD4^+^ T cell; CD8, CD8^+^ T cell; B, B cell; NK, natural killer cell; mDC, myeloid dendritic cell; pDC, plasmacytoid dendritic cell; MPP, multipotent progenitor; LMPP, lymphoid‐primed multipotent progenitor; CMP, common myeloid progenitor; CLP, common lymphoid progenitor; GMP, granulocyte–macrophage progenitor; MEP, megakaryocyte–erythroid progenitor. Figure adapted from Corces *et al* (2016). (B) Quantitative depiction of hematopoietic hierarchy, in which erythroid commitment is the predominant and default pathway of differentiation. Figure adapted from Boyer *et al* (2019). (C) Visualization of hematopoietic hierarchy in which lineage commitment occurs on a continuum rather than in punctuated stages, a perspective motivated by recent single‐cell transcriptomic studies. Figure adapted from Grootens *et al* (2019).

Every second, each one of us produces millions of diverse circulating blood cells—including erythrocytes, platelets, and leukocytes—through the coordinated process of hematopoiesis (Fig 1A–C). This dynamic cascade, by which self‐renewing stem cells that originate in the embryo go on to generate committed progenitors for the erythroid, megakaryocytic, granulocytic, monocytic, basophilic, eosinophilic, or lymphoid lineages over the course of a lifetime, is one of the best characterized paradigms of cellular differentiation (Orkin & Zon, 2008). However, our understanding of the regulation of hematopoiesis, mediated by transcription factors (TFs), cytokines, and other molecules, remains incomplete and continues to evolve (Jacobsen & Nerlov, 2019).

Early advances in the genetics of hematopoiesis were largely facilitated through the study of model organisms, including mice and zebrafish. These animal models allowed us to characterize gene function through reverse genetic approaches, with knockout experiments having a particularly prominent role over many decades. For example, much of our understanding of the Gata1 TF and its critical role in erythropoiesis stem from initial studies in mice over two decades ago, in which mice deficient in *Gata1* were found to have defective erythropoiesis (Pevny *et al*, 1991; Fujiwara *et al*, 1996). In a similar fashion, mice lacking critical hematopoietic cytokine receptors, such as the erythropoietin receptor (EpoR; Wu *et al*, 1995) or the granulocyte colony‐stimulating factor receptor (Csf3r; Liu *et al*, 1996), were shown to have defective production of erythrocytes and neutrophils, respectively. This pattern of model organism‐based reverse genetic discovery has been observed for countless other molecules critical for blood cell production (Orkin & Zon, 2008).

Although model organisms enable crucial insights into the functions of specific genes, there are important limitations to translating these findings to human biology and clinical impact. First, in contrast to the binary outcome of knockout models, most human diseases involve a diverse array of allelic variants that tune gene function or expression across a continuous spectrum, thus enabling insights into hypomorphic and other variant alleles. Second, model organisms are usually bred under a homogeneous genetic background. While this is useful for isolating the impact of specific genetic alterations, such isogenic backgrounds can mask the impact of genome‐wide genetic variation upon phenotypes of interest. In light of these considerations, a powerful way to gain additional insights is to examine the spectrum of human genetic variation in health and disease. Indeed, studies of human genetic variation have enabled a multitude of important discoveries in hematopoiesis and have been applied to better understand and treat a range of blood diseases.

In this review, we discuss advances in the genetics of human hematopoiesis in three main sections. We first discuss genetic studies of inherited rare blood disorders, which have provided complementary insights as model organisms into this process through major perturbations. We next review more recent studies of common genetic variation impacting hematopoiesis which have further refined our understanding of this process. Finally, we discuss emerging efforts to combine rare and common genetic studies to achieve a more holistic understanding of hematopoiesis. We describe several clinically relevant vignettes to illustrate how human genetic studies have revealed new knowledge about human hematopoiesis. However, we note that we cannot comprehensively cover every example and instead highlight representative examples. Finally, we look ahead and discuss outstanding questions that will guide the next decade of research in this field.

## Framework of human genetic studies

Human genetic studies can be broadly divided into common (allele frequency > 1%) and rare (allele frequency < 1%) variant association studies, each employing different approaches to work up their variants of interest. Common variant association studies (CVAS) usually take the form of genome‐wide association studies (GWAS), in which individuals are genotyped using arrays that capture mostly higher‐frequency variants. Statistical analyses can then be used to determine whether each variant is associated with a continuous or binary phenotype of interest. CVAS focus on traits with polygenic architectures comprised of many variants with small individual effects and usually include a large proportion of healthy individuals in the study population. Notable current limitations of CVAS include its high multiple testing burden from evaluating millions of variants, its inability to capture a substantial portion of heritability, and the difficulty of functionally characterizing association signals (Tam *et al*, 2019).

Rare variant association studies (RVAS) often require alternative analytical methods, since single‐variant analysis can be underpowered to detect associations if the individual mutation is too rare in the study population. To counteract this, burden tests have been developed, which collapse many variants within a gene or region into a single risk score. This approach thus performs a per‐gene or per‐region association study as opposed to per‐variant association tests in GWAS (Lee *et al*, 2012; Zuk *et al*, 2014). Another important difference is that GWAS typically employ single nucleotide polymorphism (SNP) arrays to directly genotype up to a few million common variants. Millions of additional variants can then be inferred via imputation, which is the process of using linkage patterns in a more densely sequenced reference panel to predict unobserved genotypes in the study dataset. However, these methods are ineffective for identifying extremely rare variants, especially when the variants are previously unreported or in low linkage with other variants (preprint: Van Hout *et al*, 2019). Therefore, RVAS typically use targeted sequencing, whole‐exome sequencing (WES), or whole‐genome sequencing (WGS), which allow for unbiased variant calling to identify rare or novel variants that would not have been included on genotyping arrays or that are not confidently imputed (preprint: Wainschtein *et al*, 2019). In addition, RVAS study populations are usually smaller than in CVAS and are more enriched for disease cases. Finally, some limitations of RVAS are that they usually miss non‐coding associations due to exclusion (WES) or low sequencing depth (WGS), and they require assumptions about the underlying genetic model when aggregating variants (Lee *et al*, 2014). Keeping in mind this broad framework of CVAS and RVAS, we now dive into how these approaches have been applied to study hematopoiesis in health and disease.

## Genetic studies of rare blood disorders

In the early years of human genetics, prior to the advent of high‐throughput sequencing technologies, most efforts revolved around studying rare blood diseases displaying Mendelian or monogenic inheritance patterns, and this continues to be a powerful approach today. What have such studies of rare blood disorders taught us? On one hand, they have demonstrated how allelic variation in known hematopoietic regulators creates more diverse clinical manifestations compared to the all‐or‐none knockout studies of model organisms. Secondly, they have revealed how fundamental biological processes can often have distinct and unexpected roles in hematopoiesis. In this section, we describe genetic approaches for studying rare blood diseases and then highlight examples of important biological insights gained from such studies.

Several methods have been employed to map rare blood diseases to causal genetic mutations. In the past, the most common approach was linkage analysis. This approach involves recruiting families with a disease or phenotype of interest, detecting co‐segregation of the disease with genetic markers of known chromosomal location, and pinpointing a mutated gene in the linkage window. However, since the development of massively parallel sequencing in the last decade, targeted sequencing, WES, and WGS have emerged as far more scalable and higher‐resolution ways to dissect the genetics of rare blood disorders. These approaches have had great success in identifying rare loss (or gain)‐of‐function coding variants segregating within families with hematologic traits at extremes of the phenotypic distribution (Minelli *et al*, 2004; Shiohara *et al*, 2009; Albers *et al*, 2011; Sankaran *et al*, 2012).

Studies of Diamond‐Blackfan anemia (DBA) nicely illustrate how rare variant genetics have illuminated our understanding of human hematopoiesis and how this process can be perturbed in disease in unexpected ways. DBA is an inherited hypoplastic anemia in which erythroid precursors and progenitors are selectively reduced in the bone marrow, while other lineages are ostensibly produced normally (Nathan *et al*, 1978). The first gene mapping studies used linkage analysis of families with DBA to localize a disease‐associated region to 1 Mb on chromosome 19 (Gustavsson *et al*, 1997), which was later found to be attributable to loss‐of‐function mutations in ribosomal protein (RP) gene *RPS19* (Draptchinskaia *et al*, 1999). Subsequent studies identified at least 25 additional RP mutations that explained up to 80% of DBA cases (Landowski *et al*, 2013; Ulirsch *et al*, 2018). However, how heterozygous loss of function of ubiquitously expressed RP genes could cause a selective absence of erythroid cells remained a mystery. New gene discoveries, facilitated by broader methods for genetic interrogation, enabled further insights into this disease. WES of patients who had a clinical diagnosis of DBA, but no known pathogenic mutations, revealed mutations impairing the production of GATA1 in several patients (Sankaran *et al*, 2012; Ludwig *et al*, 2014). Building upon this knowledge, subsequent functional studies solidified the link between RPs, GATA1, and defects in erythropoiesis by showing that RP haploinsufficiency reduces ribosome levels and thus results in reduced *GATA1* mRNA translation (Ludwig *et al*, 2014; Khajuria *et al*, 2018). Therefore, DBA genetics revealed new information about the regulation of GATA1 expression in human hematopoiesis, establishing a novel link between ribosome levels and GATA1 driven by its high translation rate (Khajuria *et al*, 2018).

Another example in which RVAS has enhanced our understanding of a known hematopoietic regulator is exemplified by human variation impacting *RUNX1*. Germline mutations leading to *RUNX1* deficiency cause familial platelet disorder with predisposition to myeloid leukemia (FPDMM). In 1999, linkage analysis of six separate families with FPDMM revealed that all pedigrees contained heterozygous mutations in *RUNX1* (Song *et al*, 1999). Further analysis of the affected individuals showed a deficiency in megakaryocyte colony formation, implicating *RUNX1* as a regulator of megakaryopoiesis. These cases were particularly intriguing because of the high rate of myelodysplastic syndrome (MDS) and acute myeloid leukemia (AML) in affected individuals, and demonstrated a key link between *RUNX1* haploinsufficiency and predisposition to malignant hematopoiesis (Owen *et al*, 2008). Given the challenges of studying *Runx1* in mice due to early embryonic lethality (Ichikawa *et al*, 2004), human genetic studies of FPDMM have provided powerful insights into the roles of the RUNX1 TF in normal and malignant hematopoiesis.

Additionally, RVAS of blood disorders have shown how mutations in a single master TF can result in pleiotropic and variable phenotypes, as nicely represented by the study of disorders attributable to deficiency of *GATA2*. In the past decade, researchers have found that GATA2 deficiency can cause a constellation of disparate disorders, including cases of monocytopenia with susceptibility to atypical mycobacterial infection (“MonoMAC”); loss of dendritic cells, monocytes, B, and natural killer (NK) cells (DCML deficiency); and familial MDS and AML (Dickinson *et al*, 2011; Hsu *et al*, 2011; Ostergaard *et al*, 2011). Common across this spectrum of manifestations is the notable evolution of symptoms with age, suggesting that variation in early hematopoietic stem and progenitor function may underlie many of the pleiotropic phenotypes in this disorder (Collin *et al*, 2015). Further studies of these disorders will likely provide more insights into how a master TF of hematopoiesis can lead to such disparate and variable phenotypes.

While rare variant genetics have done much to further characterize factors with known roles in blood cell production, such studies have also connected previously unappreciated molecular pathways with hematopoiesis. For example, extensive work on DBA genetics shed light on the connection between the seemingly distinct pathways of ribosome regulation and erythroid lineage commitment. There are several additional examples of this trend. For instance, investigating cases of thrombocytopenia‐absent radius (TAR) syndrome identified biallelic mutations in *RBM8A*, which encodes the Y14 subunit of the exon‐junction complex (Albers *et al*, 2012). This revelation linked a general splicing factor to hematopoiesis and suggested that lineage‐dependent deficiency of a ubiquitous protein may cause a very specific phenotype. While the exon‐junction complex has been shown to play an important role in regulating RNA through alternative splicing and may be involved in fine‐tuning gene expression (Michelle *et al*, 2012; Ishigaki *et al*, 2013; Mao *et al*, 2016), the exact basis of the mechanistic connection between the exon‐junction complex and platelet production remains unresolved. Studies of Fanconi anemia (FA) have similarly unearthed a previously uncharacterized connection between genetic mutations underlying FA and critical DNA damage repair pathways. In particular, the FA pathway has been found to play critical roles in DNA inter‐strand cross‐link repair, homologous recombination, and nucleotide excision repair, among other pathways (Ceccaldi *et al*, 2016; Sumpter & Levine, 2017; Niraj *et al*, 2019). Finally, congenital dyserythropoietic anemia type II (CDA II) was found to be caused by mutations in SEC23B, a ubiquitous component of the secretory COPII coat protein complex involved in Golgi trafficking (Schwarz *et al*, 2009), due to the absence of the paralog SEC23A within the erythroid lineage (Khoriaty *et al*, 2018). All of these examples demonstrate the broad impact of rare variant studies on advancing our understanding of human hematopoiesis and associated fundamental biological processes. There are many more examples of RVAS elucidating new pathways in diverse hematopoietic lineages that we are unable to explore here due to space constraints.

While many of the examples discussed above emerged through traditional family‐based linkage or sequencing analyses, as larger cohorts of rare disease patients are being assembled, broader assessments through RVAS and gene burden analyses are occurring. Such approaches have been valuable in the context of DBA (Ulirsch *et al*, 2018), as well as for studies of patients with rare congenital forms of thrombocytopenia and immunodeficiencies (preprint: Downes *et al*, 2018, preprint: Thaventhiran *et al*, 2018). There is no doubt that as larger collaborative efforts are established for rare disease patients, including those with genetic blood disorders, there will be more opportunities to identify additional causal and modifier genes. Moreover, these studies highlight the incomplete penetrance or variable expressivity of many alleles when examined in large cohorts of patients compared with healthy population controls (Ulirsch *et al*, 2018). Such discoveries will pave the way for further insights into human hematopoiesis.

## Population‐based genetic studies of hematopoiesis and their translation to clinical impact

In addition to the lessons gleaned from studying rare variants, there has been an equally fertile ground on the opposite side of the frequency spectrum. In this section, we will explore a burgeoning array of approaches applied to dissect *common* genetic variation and how they have advanced our understanding of human hematopoiesis.

At the population level, there is a wide spectrum of variation in commonly measured blood traits such as hemoglobin levels and blood cell counts. These traits not only cause disease at extreme ends of the spectrum (e.g., anemia, polycythemia, thrombocytopenia, and neutropenia), but also are independent risk factors for a multitude of non‐hematological diseases, including leukocyte count for coronary heart disease (Ensrud & Grimm, 1992; Hoffman *et al*, 2004) and eosinophil count for asthma (Astle *et al*, 2016), highlighting the importance of better understanding how hematopoiesis is regulated. Large family studies have estimated these blood indices to be highly heritable (Pilia *et al*, 2006), meaning that a significant portion of the observed variation in phenotype can be attributed to genetic factors. However, the precise genetic variants responsible for this variation and their mechanisms of action remain poorly understood.

**Figure 2:**
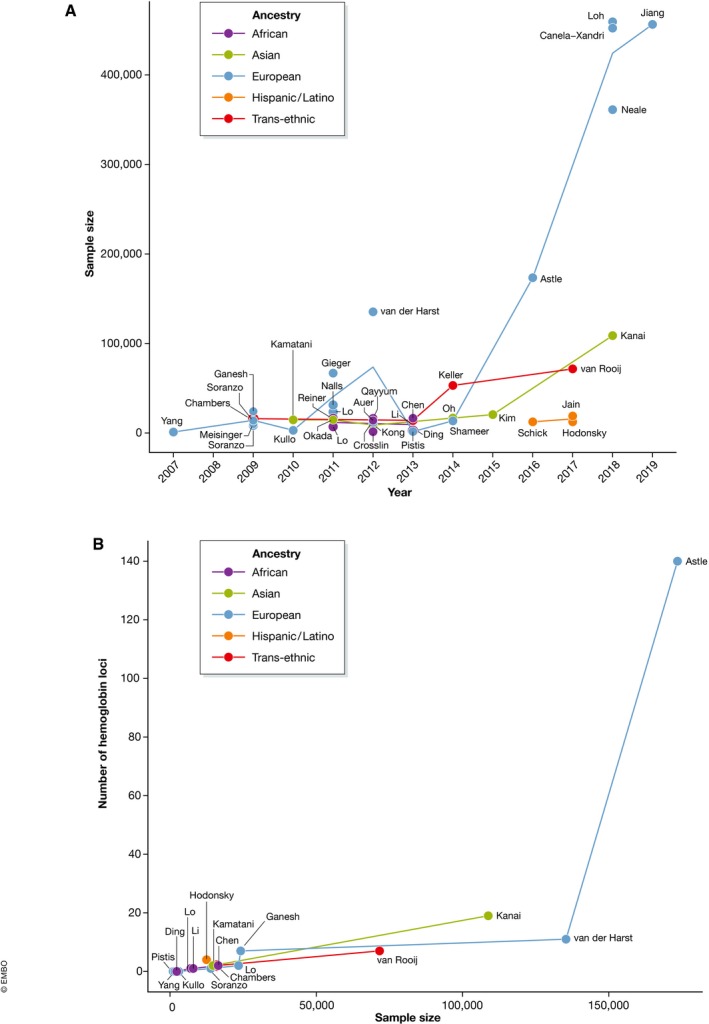
Trends in genome‐wide association studies (GWAS) of blood traits (A) Sample size of GWAS for commonly measured hematopoietic traits, including red cell, platelet, and leukocyte traits, over time. (B) Number of independent genome‐wide significant loci discovered for the hemoglobin trait as a function of study sample size. In both panels, the colors of lines and points indicate the ancestry of the study population. The text labels denote the first author of each study.

To answer these questions, many groups have leveraged natural variation in blood cell traits in healthy populations to study their genetic underpinnings, most often through GWAS. In part due to the low cost and widespread availability of blood count measurements, many large‐scale GWAS have been performed on these traits in various ancestries. Together, these studies have identified thousands of genomic loci linked to blood cell measurements (Table EV1; Fig 2A and B).

**Figure 3:**
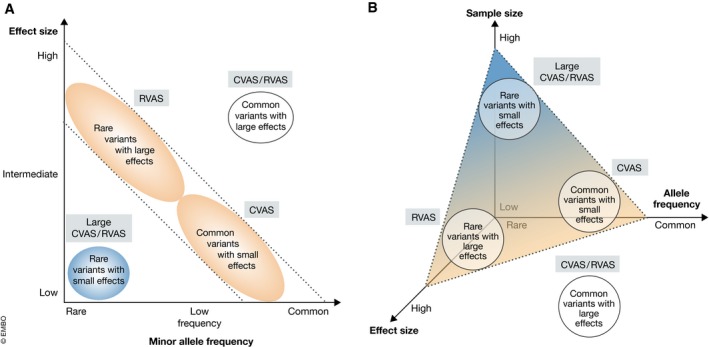
Discovery of association signals across the allelic frequency spectrum (A) Traditional depiction of variant discovery power by genetic association studies, as a function of variant effect size and allele frequency. Figure adapted from Manolio *et al* (2009). (B) Revised schematic that illustrates the 3‐way relationship between (i) sample size of the study, (ii) effect size of a genetic variant, (iii) and allele frequency of the variant on discovery power. The dashed triangular plane indicates the sample size threshold above which studies are sufficiently powered to detect variants at any given coordinate of allele frequency and effect size. The labeled circles depict categories of variants which are most often studied by the analytical methods listed next to them: common variant association studies (CVAS) and/or rare variant association studies (RVAS).

Interestingly, multiple GWASs have been performed on the same hematopoietic phenotypes over the past 15 years, with each successive study featuring more and better resolved genetic associations. Why has this been the case? Statistical power to detect true genetic associations is a function of variant allele frequency, the effect size of a variant on the phenotype of interest, and the sample size of the study (Fig 3A and B; Skol *et al*, 2006). Of these, sample size is the most scalable and extrinsic to the variant–phenotype relationship and thus has seen the largest uptick. Other advances contributing to GWAS power and resolution include the generation of larger reference panels and more accurate computational algorithms for imputation, as well as improved statistical models for genetic association testing that correct for population stratification and relatedness. These developments have collectively fueled an explosion in the number of associations identified by GWAS, including those linked to blood cell indices. For example, a study in 2009 identified a single locus associated with mean platelet volume, explaining ~ 1.5% of the total trait variance (Soranzo *et al*, 2009). Seven years later, a GWAS with a > 10‐fold increase in sample size discovered 294 significant loci, including many with lower allele frequency and smaller effect sizes, collectively explaining ~ 30% of phenotypic variance in the same trait (Astle *et al*, 2016).

**Figure 4:**
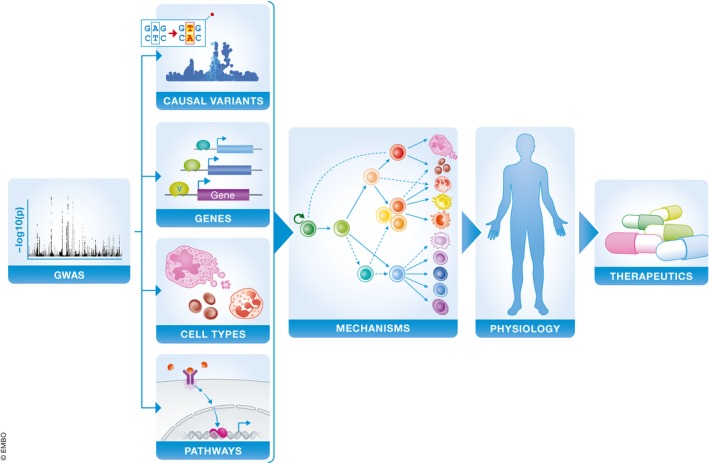
Schematic of moving from variant to function in human genetics research Blue boxes indicate key areas involved in characterizing and applying the biological mechanisms of genetic associations.

However, as a result of the rapid progress in this field, a critical follow‐up question has arisen: How can we obtain meaningful biological insight from so many robust genetic signals? In the post‐GWAS era, the challenge has shifted to not only identify genetic regions associated with blood cell traits, but also pinpoint the exact variants driving each signal, the genes targeted by these variants, and the cell types in which they act, in order to ultimately better understand mechanisms underlying the regulation of hematopoiesis in health and disease (Gallagher & Chen‐Plotkin, 2018). In other words, there is now a pressing need to move from variant to function to achieve the same richness of biological insights that have been derived from studies of rare blood diseases, as discussed above. A variety of computational and experimental approaches have been developed to tackle this multifaceted challenge (Table EV2 and Fig 4).

An estimated 80–90% of GWAS loci tag non‐coding regions, where many variants tune gene expression by modulating regulatory elements such as promoters and enhancers (Gusev *et al*, 2014). Many methods have leveraged this connection to map variants to target genes and biological pathways in a tissue‐specific manner. For example, expression‐quantitative trait locus (eQTL) studies identify variants which influence gene expression (GTEx Consortium *et al*, 2017); chromatin immunoprecipitation (ChIP)‐seq can profile DNA methylation, histone modification, and TF binding sites, which can be used to predict the potential for non‐coding variants to perturb molecular phenotypes (Chen *et al*, 2016); and chromatin capture methods identify looping interactions between enhancers and gene promoters (Javierre *et al*, 2016). High‐throughput gene knockdown or knockout screens have also become a popular experimental approach to identify genes that are important for a phenotype of interest. These screens can be implemented with short hairpin RNA or CRISPR‐Cas9 genetic perturbation to systematically test which genes or regulatory elements are essential for a phenotype, such as hematopoietic lineage differentiation (Canver *et al*, 2017; Nandakumar *et al*, 2019).

GWAS of blood cell traits, combined with these functional analyses, have provided tremendous value for understanding the transcriptional regulatory mechanisms underlying hematopoiesis. For example, such studies have identified blood trait‐associated variants which predispose or protect from clinical disease (e.g., eosinophil‐associated loci increasing risk of rheumatoid arthritis), enabled the discovery of genes and TFs important for the stage‐specific regulation of hematopoietic differentiation, and provided insights into how distinct hematopoietic lineages can be co‐regulated by pleiotropic variants acting in multipotential progenitor populations (Astle *et al*, 2016; Ulirsch *et al*, 2019).

Having reviewed the major methods used in CVAS, we now present a vignette of how these technologies have been applied to advance our understanding of a key and clinically relevant aspect of human hematopoiesis: fetal hemoglobin regulation and switching. Fetal hemoglobin (HbF) has been shown to be a key modifier of the major β‐hemoglobin disorders, sickle cell disease, and β‐thalassemia, where it is able to ameliorate symptoms through replacement of the mutated adult β‐hemoglobin (Sankaran & Weiss, 2015). Although HbF was found to be highly heritable (Garner *et al*, 2000), little was known about its precise genetic modifiers. In late 2007 and 2008, two GWAS in non‐anemic individuals identified three loci associated with variation in fetal hemoglobin levels (Menzel *et al*, 2007; Uda *et al*, 2008). These loci were also shown to be important in ameliorating the severity of symptoms in patients with sickle cell disease and β‐thalassemia (Lettre *et al*, 2008; Uda *et al*, 2008). Among these was a locus on chromosome 2 within the *BCL11A* gene, which had been well studied for its role in B lymphopoiesis and neurodevelopment, yet whose role in hemoglobin switching had not been appreciated. As a result, initial functional studies revealed a key role for BCL11A in silencing of HbF (Sankaran *et al*, 2008). In addition, BCL11A was shown to be a critical regulator of fetal hemoglobin switching in humans and mice (Sankaran *et al*, 2009; Xu *et al*, 2011). Recent studies of rare individuals haploinsufficient for BCL11A have provided additional insights into its critical *in vivo* role in silencing HbF in humans (Basak *et al*, 2015; Dias *et al*, 2016). These findings have led to a considerable effort to target BCL11A to achieve HbF induction in patients with the β‐hemoglobin disorders. These efforts include gene therapy‐based delivery of shRNAs targeting BCL11A and efforts to target an erythroid enhancer of BCL11A using genome editing approaches (Esrick & Bauer, 2018). In addition, groups have begun to perturb this pathway and identify more broadly effective therapeutic approaches for HbF induction in these diseases. Collectively, the study of HbF regulation has encompassed the full journey from variation to function and has now moved into the translational realm in an attempt to cure human diseases.

## Bridging rare and common genetics to study hematopoiesis in health and disease

There has traditionally been a division between RVAS (and other rare variant studies) and CVAS, when in reality, there is likely a continuum of genetic effects in human traits (Katsanis, 2016). Any “monogenic” disease likely acts on top of a complex, polygenic structure of variation that confers varying individual severity and risk, and any complex trait can be altered by rare, high‐effect mutations. Fortunately, on the heels of major technological and computational advances, we now arrive at an exciting crossroads in which we can begin to appreciate this full allelic spectrum underlying human variation, as the statistician Ronald A. Fisher first provided a theoretical basis for over a hundred years ago (Visscher & Goddard, 2019). In complex traits such as blood traits, schizophrenia, and autism, most of the heritability appears to be due to common alleles (Gaugler *et al*, 2014; Schizophrenia Working Group of the Psychiatric Genomics Consortium *et al*, 2014), yet recent large‐scale sequencing studies have uncovered a concomitant enrichment in rare loss‐of‐function variants in these and other phenotypes (Chami *et al*, 2016, preprint: Karczewski *et al*, 2019; Weiss *et al*, 2008), suggesting an interplay between rare and common variants. Moreover, in other complex traits including height and body mass index (BMI), WGS studies suggest that variants with low minor allele frequency explain a substantial portion of the total heritability that has not been able to be explained by common variants alone (preprint: Wainschtein *et al*, 2019).

The same convergence can also be observed in other areas of the hematopoietic system. For example, despite being defined as a monogenic disorder by a coding mutation in the β‐globin gene, sickle cell disease (SCD) exhibits extensive clinical heterogeneity, ranging from mild phenotypes that remain undetected for decades to severe forms with multiorgan damage and early mortality (Platt *et al*, 1991). Two of the strongest modifiers of SCD severity are HbF levels and white blood cell count (Miller *et al*, 2000; Steinberg & Sebastiani, 2012; Bao *et al*, 2019). Rare and common variants are known to be independently associated with variation in these traits (Galarneau *et al*, 2010), and consequently influence SCD severity. Similarly, in β‐thalassemia, a disorder defined by mutations affecting the expression of the *HBB* gene, genetic studies have found that common variants influencing HbF at least partially explain the significant variation in disease severity (Nuinoon *et al*, 2010). Both of these cases highlight the importance of studying “monogenic” diseases in the context of common genetic variation.

Another example of this convergence can be seen in mutations altering GATA1 activity or function both globally and at specific loci. On one hand, GATA1 bound cis‐regulatory elements (CREs) are frequently impacted by common genetic variation associated with hematopoietic traits, but these variants primarily tune the activity of such CREs with effect sizes below what is required to cause frank disease (Ulirsch *et al*, 2016). On the other end of the spectrum, mutations disrupting the GATA1 motif in critical CREs can cause a variety of monogenic blood disorders due to impaired gene expression (Manco *et al*, 2000; Campagna *et al*, 2014; Kaneko *et al*, 2014; Wakabayashi *et al*, 2016). Moreover, mutations in GATA1 itself can result in a range of phenotypes that include a complete absence of erythropoiesis, as is the case in DBA, or more subtle defects in red blood cell and platelet production (Crispino & Horwitz, 2017; Abdulhay *et al*, 2019). Putting these disparate findings together, one can theorize that these disease‐causing *GATA1* mutations do not exert their effects in isolation, but rather act on top of a complex, polygenic structure of genetic variation that confers varying predisposition to alteration in blood cell traits (e.g., CREs at specific loci may be more or less sensitive to disruption of key master TFs, like GATA1). Only by considering this complete spectrum of allelic variation can we begin to explain all of the nuances and variation observed in blood disorders.

What mechanisms could mediate this phenomenon in which common variants influence rare disease mutations? Most genetic studies assume an additive effects model, whereby independent risk alleles contribute to a uniform, linear increase in an associated phenotype. From this perspective, individuals who harbor risk alleles across common variants tuning blood production, combined with a rare disease‐causing mutation, could manifest with a particularly severe phenotype. However, the biology can also be more complicated. Epistasis refers to interactions between loci in their effect on a trait, such that the impact of a particular genotype depends on the genotype at other loci in a non‐independent manner (Wei *et al*, 2014). For example, in a number of rare monogenic disorders, the deleteriousness of the disease‐causing mutation can be modulated by neutral or benign alleles in the same haplotype (Jordan *et al*, 2015) or at distinct, but molecularly related, loci (Timberlake *et al*, 2016). Together, these additive and complex genetic interactions likely act in concert to tune the penetrance and expressivity of hematopoietic phenotypes and traits.

An important next step is to assess how well our expanding coverage of human genetic variation can predict complex traits in heterogeneous populations and serve as clinical biomarkers. Polygenic risk scores (PRSs) serve as a prime example of this endeavor. PRSs are a weighted sum of risk alleles carried by an individual, in which the risk alleles and their weights are defined by their effect sizes on a phenotype of interest. In many complex traits and diseases, PRS can already quantify risk more accurately than current clinical models (Sharp *et al*, 2019), at times identifying individuals with an increased risk equivalent to those with rare monogenic mutations (Khera *et al*, 2018). Other studies have found that combining common variant PRSs with other known modifiers, including lifestyle factors and rare mutations, can further improve prediction accuracy for disease risk and severity (Niemi *et al*, 2018). To our knowledge, there has been no large‐scale attempt to implement PRS on hematopoietic traits to date, but given the substantial heritability of these phenotypes (Pilia *et al*, 2006), their wide spectrum of continuous variation, and multifaceted connections to clinical parameters ranging from inflammation to hemostasis, blood traits are promising targets for PRS prediction.

Thus, perhaps we should shift our perspective on allelic variation underlying hematopoiesis from a dichotomous lens involving rare or common variation, to one involving a spectrum of variation that collectively impacts the process of hematopoiesis to varying extents to alter traits or cause disease. From a technical standpoint, we are now poised to fully bridge this gap. In the early years of genetic studies, sample sizes were limited such that for complex traits, only common variants could be detected by GWAS, whereas in presumed monogenic diseases, only highly penetrant and deleterious rare variants could be readily identified. Now, with increasingly large sample sizes, GWAS for common phenotypes can detect a greater proportion of rare variants with real effects, whereas rare disease studies are better powered to assess how common variants tune the penetrance of disease‐causing rare variants. Looking ahead, the future is bright for researchers to study the impact of the entire allelic variation spectrum on hematopoiesis.

## Concluding remarks and future perspectives

Modern genetic analysis has transformed our understanding of the genetic determinants of human hematopoiesis and the diseases that ensue when these processes go awry. This has enabled us to gain considerable insights beyond the valuable observations made in model organisms. Looking ahead, there is tremendous potential and excitement for the future of genetics and applications to understand human hematopoiesis. One important goal over the next 10 years is to map the full allelic spectrum of how genetic variation regulates hematopoiesis in health and disease. Furthermore, despite the enormous strides the field of genetics has already made, and a critical shortcoming has been that the majority of large studies have been confined to populations of European ancestry (Martin *et al*, 2019). Therefore, an essential part of this challenge is to expand genetic studies to populations of different ancestries. As data for underrepresented subpopulations become more available, it is likely that numerous additional population‐specific loci and variants will be uncovered.

As illustrated in this review, we have already started to bear the fruits of increasingly powered rare and common genetic association studies. Merging knowledge of how rare variants contribute to disease with our growing understanding of common variation in human genetics will allow us to more fully characterize and explain the genetic architecture of blood cell production. In addition, with powerful functional tools becoming increasingly available, we can begin to glean rich insights into human hematopoiesis from functional studies that span the allelic spectrum and enable us to move from variant to function.

### Author contributions

ELB: conception of article, article draft, literature research, and preparation of figures; ANC: conception of article, article draft, literature research, and preparation of figures; VGS: conception of article, article draft, and editing.

### Conflict of interest

The authors declare that they have no conflict of interest.

**Pending issues**

(i) Mapping the full allelic spectrum of how genetic variation regulates hematopoiesis in health and disease.
(ii) Expanding genetic studies to populations from more diverse ancestries.
(iii) Improving the resolution and scalability of efforts to characterize the functional mechanisms by which genetic variants impact hematopoiesis.
(iv) Development and implementation of genetic risk scores to improve blood trait predictions in clinical settings.

## For more information

- https://www.ebi.ac.uk/gwas/home
- https://clinicaltrials.gov/ct2/show/NCT03282656

## Supporting information



Table EV1

Table EV2
